# Supplementary material for: Comparative analysis of predictive methods for early assessment of compliance with continuous positive airway pressure therapy
Source: BMC Med Inform Decis Mak. 2018 Sep 18;18:81. doi: 10.1186/s12911-018-0657-z (PMC6145365; doi:10.1186/s12911-018-0657-z)
Supplement: Supplementary file 1 — Tables with the description of all the variables of the D0, D1 and D3 datasets. Tables with the results of the descriptive analysis of the datasets. (DOCX 944 kb) [file 12911_2018_657_MOESM1_ESM.docx]

**Supplementary material**

| 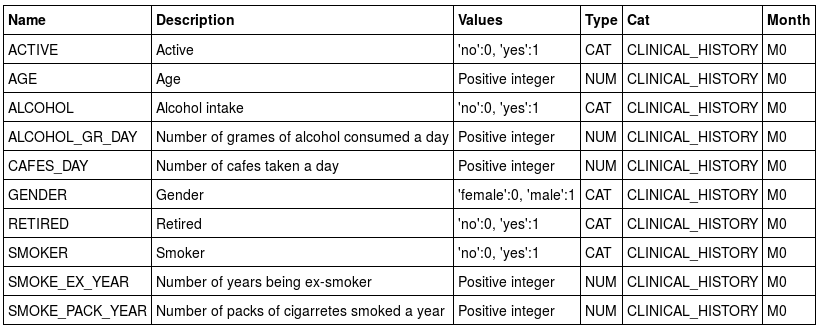  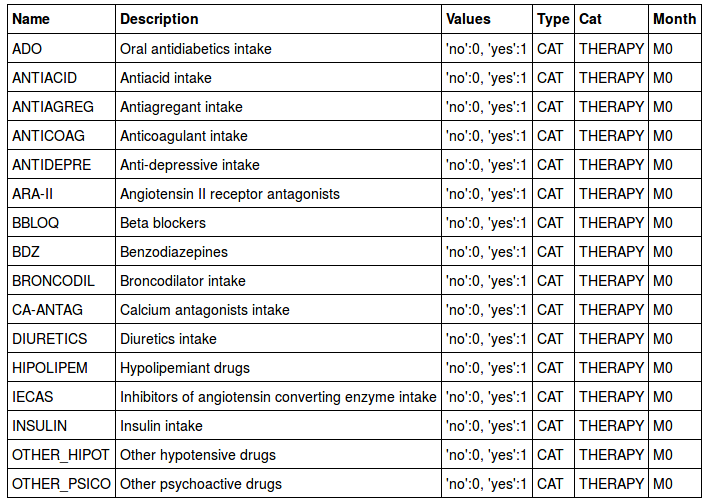  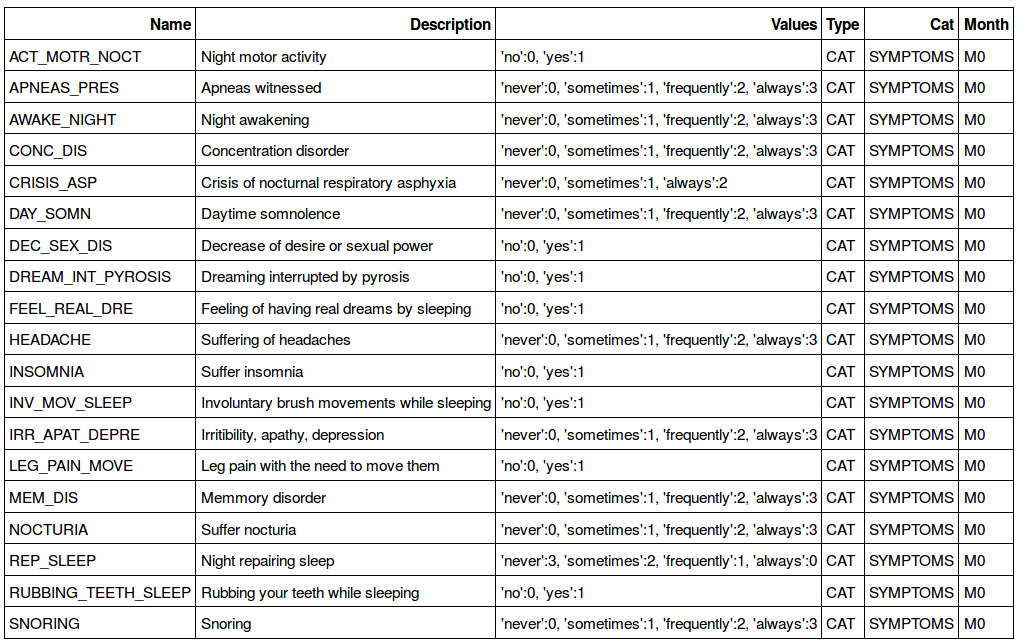  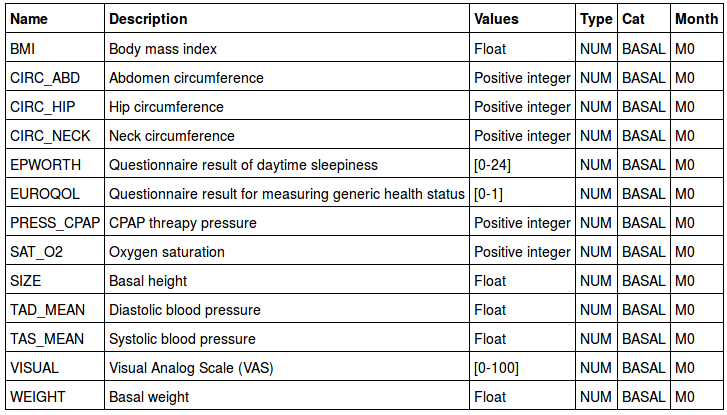  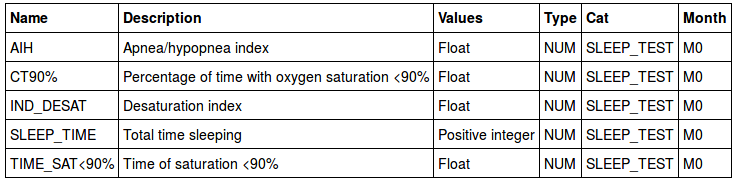  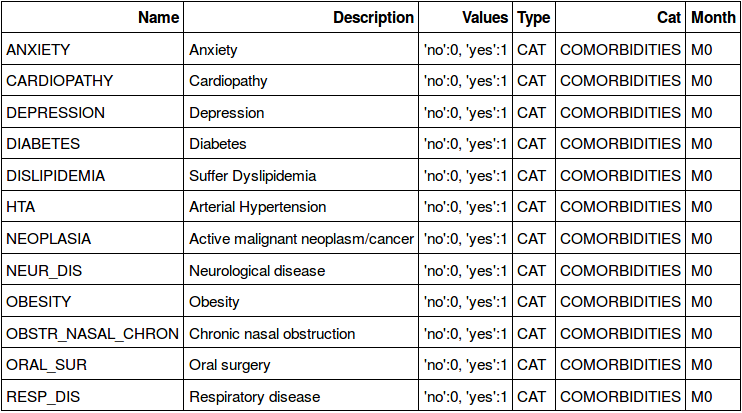  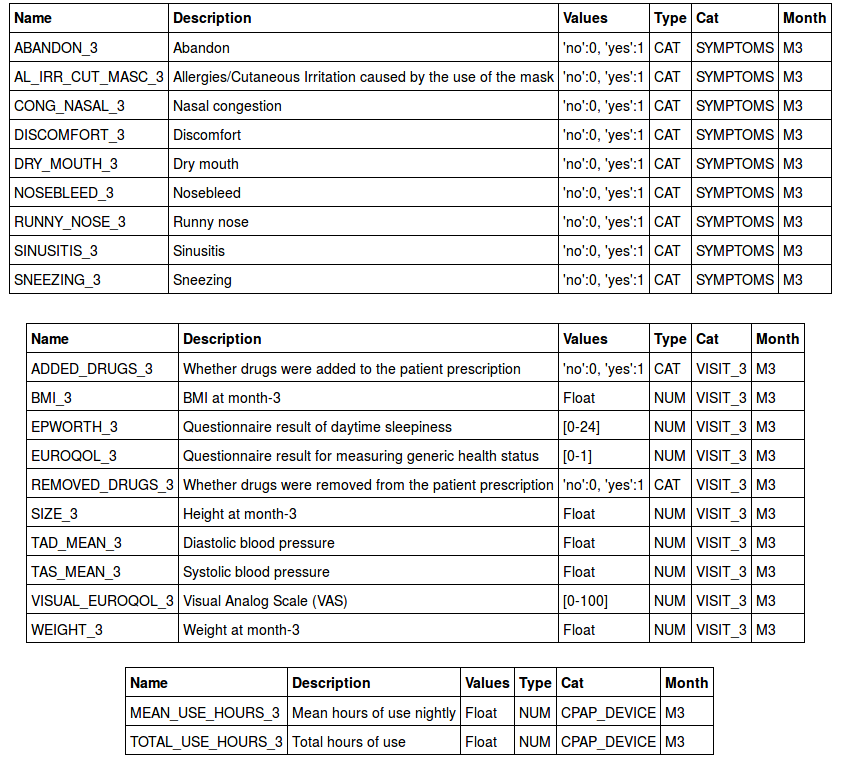  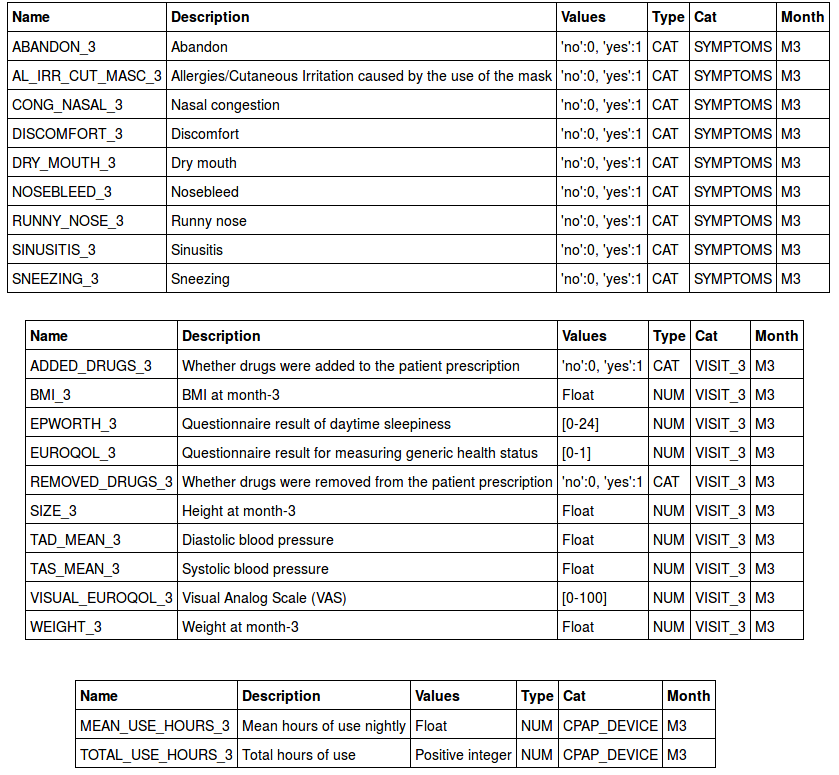 |
| --- |
| **Table s1**. List of features grouped by category type (i.e. clinical history, symptoms, therapy, sleep test, comorbidities and basal, visit_1 and visit_3) |

| 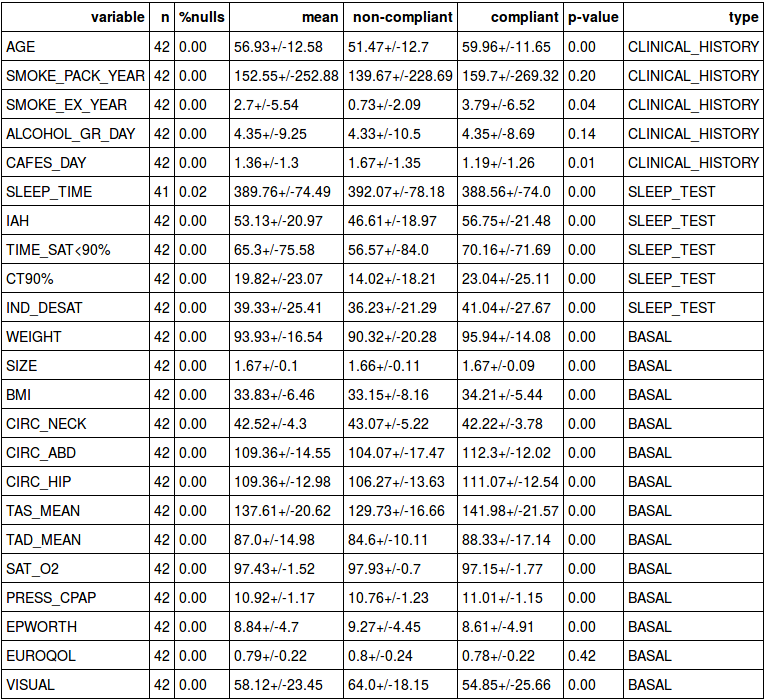  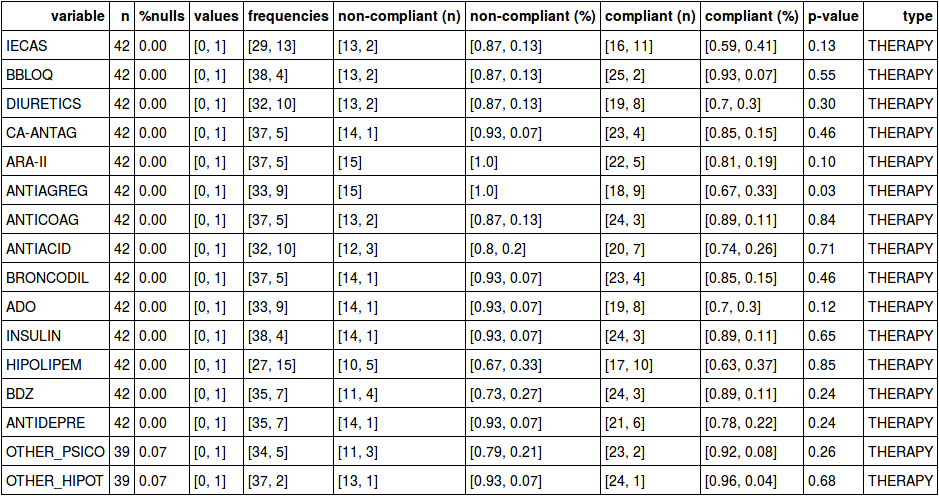  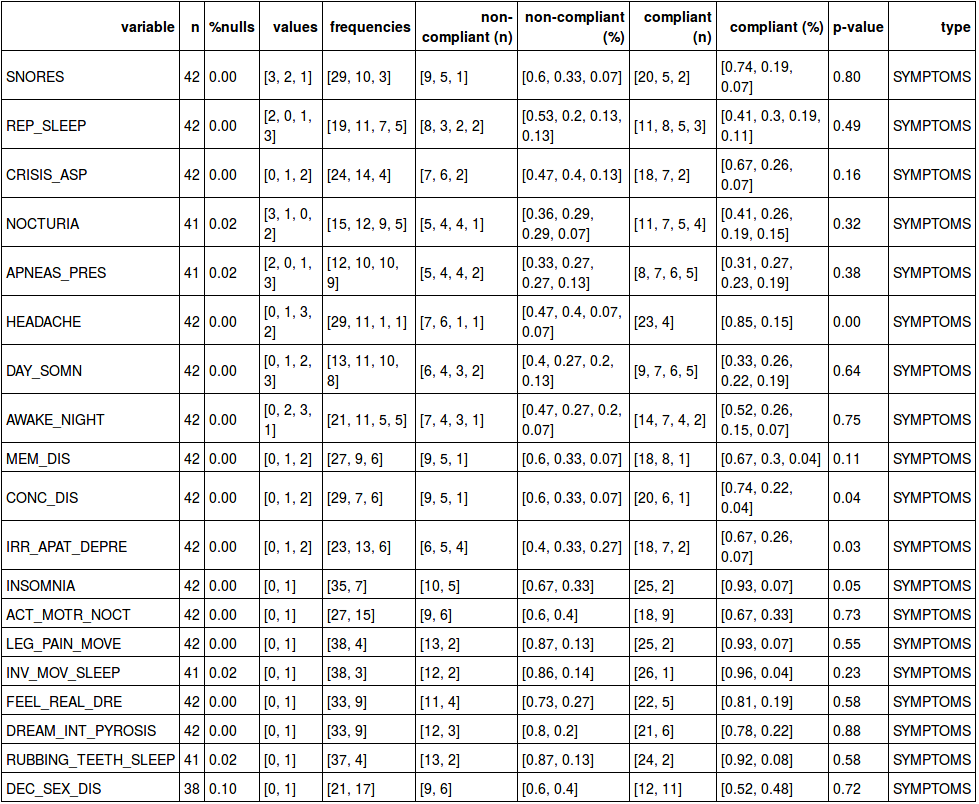  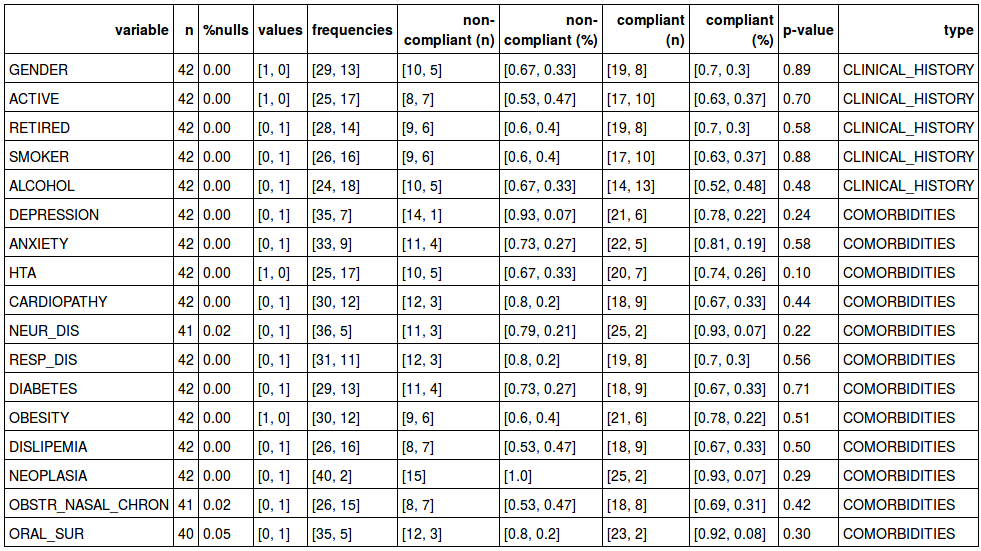 |
| --- |
| **Table s2.** List of all features at baseline (month-0) |
| 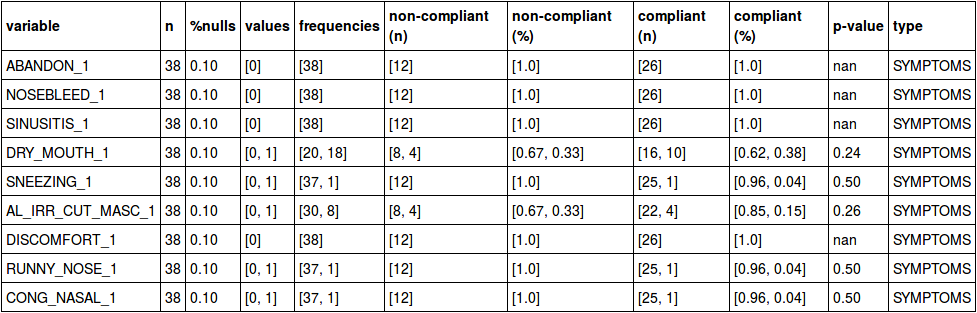  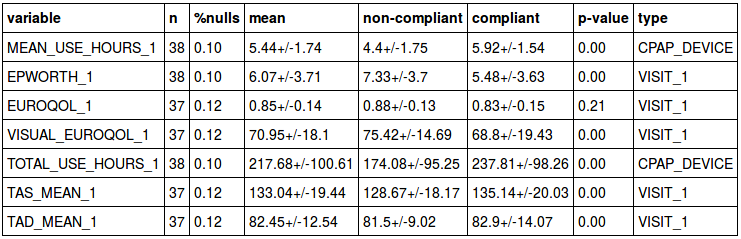 |
| **Table s3.** Features collected at month-1 |

| 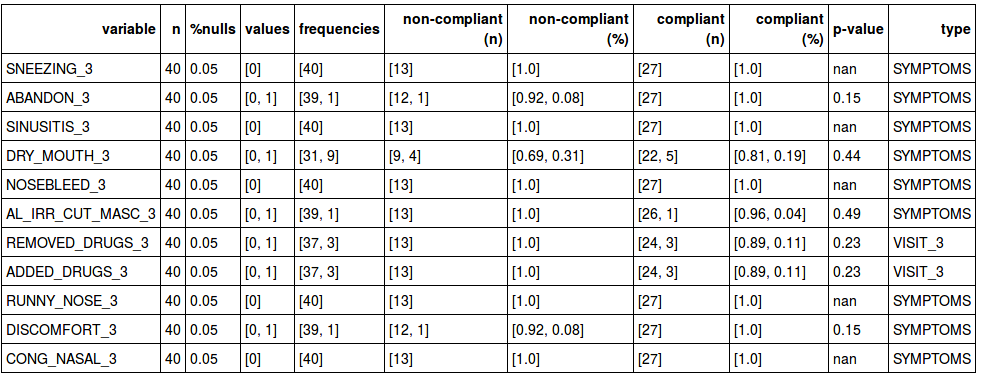  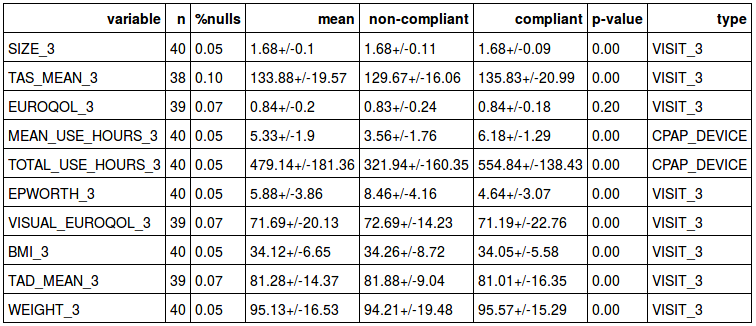 |
| --- |
| **Table s4.** Description of features collected at month-3 |
